# The Neglected Traditional Enset (*Ensete ventricosum*) Crop Landraces for the Sustainable Livelihood of the Local People in Southern Ethiopia

**DOI:** 10.1155/2022/6026763

**Published:** 2022-04-30

**Authors:** Newarinesh Feleke, Wondimagegnehu Tekalign

**Affiliations:** Department of Biology, Wolaita Sodo University, Wolaita Sodo, Ethiopia PO Box 138

## Abstract

Enset (*Ensete ventricosum*, Musaceae) is a neglected traditional multipurpose crop plant critical for Ethiopian food security. It has drawn a lot of attention in the last few years. This study was undertaken on the morphological diversity among the enset landraces and their cultural use for the livelihood of the people in Southern Ethiopia. The study was administered in four purposively selected kebeles of the Mareka District. A total of 145 individuals were interviewed using semistructured interviews, and field observation has also occurred. The descriptors for enset developed by the International Board for Plant Genetic Resources were used to measure the morphological features. This study found twenty-two enset landraces. Landraces were categorized into five groups based on their morphological trait variability. The highest mean was in cluster five, while the lowest was in cluster three. The highest landrace diversity was found in Ocha (*n* = 2.28) and the lowest in Guta (*n* = 2.17). This study confirmed that the study area has a diverse range of ecosystems. However, a reduction in production and the loss of some landraces were observed. As a result, the protection and preservation of enset landraces must be a priority for all responsible entities.

## 1. Introduction

Enset (*Ensete ventricosum* (Welw.) Cheesman) is one of the root crops, which are perennial herbaceous and monocotyledonous crops that belong to the Musaceae family and flower just once in their life cycle depending on the climate and landrace type [1]. It is closely associated with and features a physical resemblance to the banana plant, as a result of which it is sometimes referred to as a “false banana.” The crop is versatile and environmentally resilient [2]. It is currently a staple and/or costaple diet for 20 million Ethiopians, or 20% of the population [2, 3].

Being perennial, enset improves the local climate and soil conditions [4]. The *Ensete ventricosum* species is found in the wild throughout Sub-Saharan Africa and Asia [5, 6], and it originated in Ethiopia [7]. Enset is cultivated only in South and South-Western Ethiopia's native indigenous farming systems [8]. It is the main crop that ensures food security in a food-deficient country. According to Tsehaye and Kibebew [9], enset has been grown in Ethiopia for over 10,000 years. Its plant economy is one of the main agricultural activities in Southern Ethiopia. It has been reported by Tsegaye and Struik [10] that, in a comparison of starch crops, enset produces the highest yield per hectare in Ethiopia with relatively low inputs. This crop has several gastronomic, sociocultural, medicinal, ecological, and commercial benefits, and it helps rural communities achieve food security and reduce poverty [11].

Enset domestication dates back to the Neolithic period or even earlier [8, 12], and its farming system has appeared as one of the few historic and sustainable agricultural systems in Africa [13]. Traditional farmers' knowledge and practices support the generation and continued maintenance of on-farm ecosystem diversity [10, 14]. Local knowledge, experience, and cultural values play a substantial role in the sustainable management, conservation, and utilization of genetic resources and the restoration of agroecosystems [15, 16].

Current research indicates that 67 different vernacular names for enset landraces are under cultivation. There are 31 landraces in the lowland and 52 in each of the highland and midland agroecologies, with 22 shared across the three agroecologies [6]. In general, many landraces are identified by vernacular names and show a narrow and unique pattern of distribution [17]. High enset genetic variety dispensed over a huge variety of environmental situations shows that the domestication method can also facilitate the adaptation of landraces to local conditions and, indeed, to a wider range of conditions than their wild progenitors [3]. Previous research has shown that the genetic variety of enset is decreasing over time. This could be owing to farmers' prioritizing certain clones, genetic degradation, or a small sample size for the researcher.

A landrace may be defined as a variable population that features a local name, lacks formal crop improvement, and is related to the traditional uses, knowledge, habits, and celebrations of the people that developed and continue to grow it [18]. The local diversity of enset is restricted despite the use-value of the crop as food for the bulk of the people in Southern Ethiopia. This could have resulted in the extinction of existing varieties and indigenous knowledge [9].

Enset might be a multipurpose crop that uses every component of the plant. It is primarily used as a raw material for industries and construction materials and for human consumption, cattle feed, medicinal reasons, and ornamental purposes [19–21]. It enhances the local climate and soil conditions because it is perennial [4]. For many Ethiopians, the *Ensete ventricosum* food product is a staple and/or costaple food security crop and their primary energy source, particularly in the highlands of the country's southern, southwestern, and central regions, where population density is high [20, 22–24]. Furthermore, due to its high yield and drought tolerance, this plant contributes significantly to global climate change-related food insecurity in many underdeveloped countries [20, 25].

Kocho, bulla, and amicho are the most common dishes made from enset, and these processed enset products are high in carbs and minerals [19]. It does, however, lack proteins and vitamin A [22, 26]. The pulp of the pseudostem, the immature shoots, and the corm are all edible sections of the enset, albeit the edible parts vary by region. When the processed pseudostem is fermented, it creates flour, which is then dried and used as a basic ingredient in bread and porridge [20, 22–24]. Food processing from enset is time-consuming, so technical advancements that make the job easier while maintaining food quality are required [27]. Food processing of enset is based on people's traditional expertise and differs across the country's enset-growing regions [23].

The enset plant's corm and pseudostem are traditionally processed into kocho, a key food product [23]. Fermented kocho is frequently kept in pits lined with enset leaves. The kocho must be held in a storage hole for at least a month, although it can be kept for many months, if not years [22]. Kocho is the main component of fermented starch made from scraped leaf sheaths and grated corm (underground stem base). Kocho can be preserved for a long time without becoming bad. The age of the harvested enset plant, the type of clone (variety), and the harvesting season all influence the quality of kocho. Furthermore, the amount of leaf sheath and corm treated inside a single plant affects quality. The preferred kind is white and comes from the innermost leaf sheaths and the inner section of the corm, whereas the lowest grade is blackish and comes from the outside leaf sheath and corm [20]. Kocho, a fermented enset bread, has grown more popular in Ethiopian eateries that serve kitfo (raw ground beef combined with butter and spices) [19]. At restaurants, the combination of kocho and kitfo is now practically mandatory [24]. Bulla is made by pulverizing the leaf sheath, peduncle, and grated corm; squeezing the starch-containing liquid from the pulp; and enabling the resulting starch to concentrate into a white powder by evaporating the water and rehydrating with water. The highest-quality enset meal is derived primarily from fully matured enset plants. Bulla is a pancake, porridge, or dumpling that can be made in a variety of ways [19, 20, 28]. A boiling enset corm, usually from a younger plant, is called an amicho. If the amount of enset harvested is insufficient, or for special events, enset plants can be removed to prepare meals quickly. The corm is boiled and eaten the same way as other roots and tuber crops. Certain clones are chosen for their ability to produce amicho [8, 19, 20].

This study aims to identify the prevailing enset landrace diversity, morphological trait diversity among enset landraces, threats to enset diversity, and associated indigenous knowledge of the people in the study area. The ultimate goal is to gather information that will aid in establishing a scientific foundation for the plant's maintenance and use. The ultimate goal is to provide information that will aid in the development of a scientific foundation for the plant's long-term maintenance and use.

## 2. Materials and Methods

### 2.1. The Study Area

**Figure 1:**
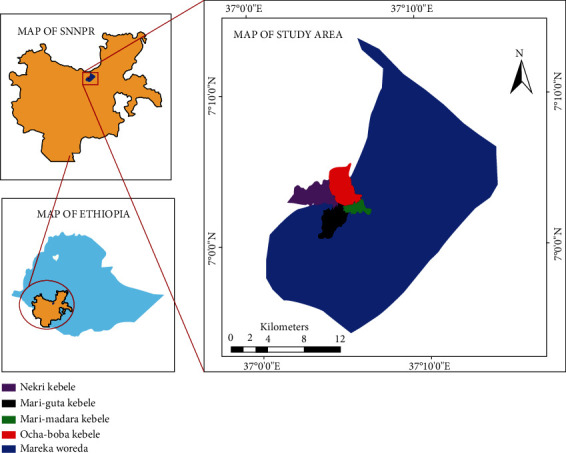
Map of the study area.

The field sites for this study were Ocha Boba, Nekir, Mari Guta, and Mari Madara kebeles (the smallest administrative units) of the Mareka woreda in the Dawuro zone, SNNPRS. Its geographical position is between 37° 0° and 37° 1° E latitude and 7° 0° and 7° 1° longitude (Figure 1). It is situated 438 km southwest of Addis Ababa, Ethiopia. Enset is their main staple crop, but other cash crops are also grown. Animal husbandry is practiced but mainly used for milk supply and dung. The annual maximum temperature of the study area ranges from 22.4 to 28.3°C, while the mean annual rainfall ranges from 976 to 1404 mm.

### 2.2. Methods

The study sites were selected to support the areas with high production of enset and individual enset landraces that play economic and cultural roles. The areas were selected by referring to literature sources, the survey made by CSA [29], the production of major crops, and using the suitability map of enset on the crop's ecological requirements [30]. Information about the production of enset was taken from the district agricultural office. Random selection methods selected the households. A total of 145 households were selected from four kebeles (Ocha = 46, Nekir = 33, Mari Madara = 38, and Mari Guta = 28) (Table 1).

The sample sizes of households were determined by using Yamane's [31] formula.

(1) $$\begin{array}{c} n=\frac{N}{1+N\left(e\right)2}, \end{array}$$

where *N* = the total population studied*n* = required sample size*e* = the precision level, which is (0.08%) where the confidence level is 95% at *p* = ±5 (maximum variability)

Ethnobotanical data was gathered in order to better understand farmers' indigenous knowledge of enset plants. Different qualitative and quantitative ethnobotanical data collection methods, like field observation, guided field walks, semistructured interviews, and market surveys, were used to get the participants' needed information. The following ethnobotanical information was gathered using a semistructured interview: the local name of the crop and landrace; the time of cultivation and harvesting; traditional management practices; the cropping system, uses, and market value of the crop; landraces that persist in drought, disease, and pest and have short maturity times; the planting material exchange system; production constraints; and farmers' perceptions of the crop.

To characterize the agromorphological traits of the landraces, all landraces found in the study area were measured by their quantitative and qualitative meanings. The IBPGR [32] approach was used to evaluate the fifteen agronomic characteristics of enset for each landrace (Table 2).

There were different morphological and agronomic characteristics that farmers used to identify their landraces in the study area. Some of them are the color of the pseudo-stem, midrib, leaf, petiole, time of maturity, disease resistance, yield, leaf dimensions (width and length), and pseudostem length.

### 2.3. Data Analysis

Descriptive statistics were used to analyze data obtained through interviews and guided field walks.

### 2.4. Preference Ranking

In preference ranking, 20 key informants were selected from four kebeles and were asked to rearrange a gaggle of things consistent with a given criterion like personal preference or the importance of a species. Each item was then assigned a value, with the most important or preferred item being ranked with the highest value, while the least preferred item was ranked with the lowest value. As a result, in this investigation, distinct enset species utilization values were short-listed and ranked by informants using the Martin [33, 34] approach.

### 2.5. Direct Matrix Ranking

Direct matrix ranking was applied in order to answer the question of which landrace was best for which purpose. The chosen informants reported the landraces and their purposes. Then, each key informant was asked to rank the landraces for each of the purposes. The values of every landrace were summed up and ranked for every informant and then finally for the entire informant population.

### 2.6. Morphological Diversity Analysis

Enset landrace diversity analysis, including the Shannon-Wiener Diversity Index (*H*) [35], and the richness and evenness of each study kebele were analyzed. The Shannon-Wiener Diversity Index (H) was used to analyze the phenotypic diversity of ensets depending on the traits that were measured, counted, and recorded, and the richness and evenness of each study were analyzed. It was calculated using the formula.

(2) $$\begin{array}{c} \text{H}=-\overset{\text{s}}{\underset{\text{i}=1}{\sum }}\text{p}\text{i linpi}, \end{array}$$

where *S* is the number of phenotypic classes for a character and pi is the relative proportion of the total number of entries (*N*) in the *i*^th^ class [36]. Richness is measured by the number of individuals, irrespective of their frequencies. Evenness, however, measures how similar the frequencies of the various variants are, with low evenness indicating dominance by one or a couple of types. Evenness has values between 0 and 1, where 1 indicates the condition where all landraces are equally abundant, while 0 indicates that a few landraces are more abundant. Evenness is calculated, where *H* is the Shannon-Wiener Diversity Index, *H*_max_ is ln (*N*), and *N* is the total number of landraces.

## 3. Results

The assessment on the size of the land indicated that the majority (55%) of the respondents had 2-4 hectares of their own land that was used for farming purposes, including home gardens, and the maximum amount of land owned by the respondents was 11 hectares of land. The largest recorded land cover of enset was 28% hectares and, on average, 13% hectares of land on a farm. Some of the respondents (15%) reported having 9–13 enset landraces growing in their yards, while most of the respondents (43%) grow only three to six enset landraces.

A total of 22 landraces were identified from the four kebeles of the study area (Table 3). Depending on the landraces cultivated in the home gardens, the most frequently mentioned descriptors for identification were pseudostem color (29%), midrib color (19%), plant size (12%), and leaf color (27%). The majority (58%) of the farmers lost their landraces within the last 15–25 years, while a few (12%) farmers lost their landraces before the last 25 years. The lost landraces were known as Lochingiya and Yaka. Fifty-nine percent of the farmers' interest in growing enset was decreased, 31% increased, and 12% showed no change or stability in the production of enset.

The enset landraces were grouped into five clusters based on the morphological traits (pseudostem color, petiole color, leaf color, midrib color, kocho quality, bulla quality, and fiber quality) and agronomic characteristics (disease resistance and drought resistance). Cluster one includes the most important number of enset landraces (*N* = 11). Landraces are distinguished by their light green pseudostems, deep green leaves, light green midribs, high-quality fiber, and resistance to drought and disease. These were Yesha Maziya, Hoeya, Amiya, Shasha, Yaka, Bothena, Ontha, Botha Maziya, Erantiya, Ankuwa, and Boza. In cluster two, the landraces provide high-quality bulla, kocho, and fiber. These are Arkiya Budunthuwa, Gena Shododiniya, Mataka, and Aguntha. Cluster three consists solely of Kuruwa and Wosa ayfiya landraces. They have a dark red pseudostem, deep green leaves, medium fiber quality, and are vulnerable to drought and diseases. Cluster four includes Keteriya, Tochinuwa, Chamerotiya, and Udunthiya landraces. It was well defined based on the leaf. They had purple leaves and were resistant to diseases and drought. Cluster five includes landraces having deep red pseudostems, deep red petioles, yellowish-green leaves, and high resistance to disease and drought. These are Shakariya, Adinona, Lochingiya, Kataniya, Koshikoshiya, Babaka, Badaluwa, and Wora Kana Utha landraces (Table 4).

When the diversity of enset was estimated based on the number of landraces (richness), Ocha Kebele (of the Mareka District) showed the largest richness (*H* = 2.27; *D* = 17.99), followed by Nekir Kebele (*H* = 2.24; *D* = 11.99). However, Guta kebele showed the lowest richness (*H* = 2.17; *D* = 10.59). According to the farmer, Ocha has the most landraces on average, followed by Nekir, Madara, and Guta in that order (Table 5).

In Ocha kebele, the most dominant enset landraces were Amiya, followed by Hoeya, Boza, and Botha Maziya, respectively, while in Nekir kebele, the dominant landraces were Ontha, Yaka, and Adinona. In Guta kebele, Shasha, Erantiya, Ankuwa, Gena, Budunthuwa, and Kuruwa were the dominant ones. The dominant landraces in Madara kebele were Keteriya, Arkiya, Argama, Mataka, and Shododiniya, respectively. The most widely used landraces in Ocha kebele were Amiya, Yesha Maziya, Boza, Hoeya, Bothena Ontha, Ankuwa, Shasha, Yaka, and Botha Maziya (Table 6).

The diversity of landraces in the study area was measured by the richness (*C*), evenness (*E*), Simpson (*D*), and Shannon (*H*) indices.

All enset-growing farmers are growing enset plants in their home gardens. Fifty percent of the respondents grow enset as a sole crop. They are used to control disease and pest spread and to minimize food and water competition. Thirty percent of the land was intercropped with coffee, chat, mango, avocado, and apple to maximize land use, and the remaining twenty percent was border cropped with vegetables like cabbage and tomato to protect susceptible crops from disease and pest attack and to use the enset as a windbreak.

Out of the total informants, 80% used enset only for household consumption, while 20% of them used a quarter of their enset products as a source of income generation. The enset plant is used as a major food source, utilized in different forms by the local people for their daily consumption, and it has been a means of subsistent livelihood for the community since several years ago (Table 7). All the enset landraces are prepared in the form of “kocho” food (a fermented product from scraped pseudostem and grated corm) and are prepared by scrapping the leaf sheath and grated corm, wrapped in enset, and stored underground until fermented in different forms.

Besides, it is utilized in the shape of bulla food (dehydrated juice) and is ready to scrape the leaf sheath peduncle and grated corm into a pulp, squeezing liquid containing starch from the pulp, allowing the resultant starch to concentrate into white powder and rehydrating with water. As shown in Table 8, the Arkiya, Lochingiya, Badadiya, Argama, Kataniya, Mataka, Aguntha, and Boza landraces have all been reported to treat various ailments in the study area.

The preference ranking on the use-value of enset by the key informants showed that enset is primarily used as a source of food by the local people, which is followed by its use for medicinal purposes, and therefore the least used as a means of income generation. Preference ranking based on enset use-value within the study area was indicated in ascending order from 1 (least useful) to 5 (most useful) (Table 9).

## 4. Discussion

The Abyssinian banana is found at the highest frequency among different landraces. According to the informant report, each farmer owns a number of enset landraces on their farmland. The traditional identification mechanism used by the farmers is similar to that used by the Sidama and Wolaita zones [6, 37]. In the case of maturity time, almost all landraces in the study area have the same maturity time.

Both the Botha Maziya and Boza landraces were the most disease-resistant, while the Lochingiya, Badadiya, and Kataniya landraces were the most susceptible. Botha Maziya and Ontha landraces are known for their quality of Kocho. Boza, Hoeya, and Shasha are high-yielding landraces, but they require a low level of Kocho and Bulla. According to the respondents, enset is cultivated by every household; however, its cultivation is declining from time to time. The increasing demand for engaging in the production of other crops might be the reason for the decreasing trend of enset production in the study area. This might also be due to population growth and a shortage of land, which leads them to food shortages as they are forced to grow short-season crops instead of enset. This agrees with the recent reports by Abebe and Eshetu [38] that were done on the vulnerability of agricultural systems and agrobiodiversity in Southern Ethiopia. The difficulty of the processing system might also be another reason for the reduction of enset production in the area. This result was similar to the study by Alemu and Sanford [39] that was done in the North Omo Zone, Ethiopia.

In this study, 22 enset landraces were identified. Yemataw et al. [40] and Zeberga et al. [2] recorded 27 and 53 locally known enset landraces, respectively, in different districts of the Gurage Zone, Ethiopia. The variations in the number of enset landraces recorded in different parts of the country might be due to the difference in the number of sampled zones selected for the study. The number of enset landraces (richness) per household varies from kebele to kebele. In these kebeles, the majority of the landraces were replaced by landraces that were disease resistant. Jarvis et al. [41] described how, at the scale of traditional landraces, it requires prior determination of the identity of the landraces. There was no significant difference in the number of landraces at the kebele level. The number of landraces increases as the number of surveyed households increases and becomes constant as the number of households continuously increases.

In the study area, farmers cultivate enset crops in their home gardens, followed by chat and coffee crops. A similar study by Magule et al. [6] in the Wolaita Zone, Southern Ethiopia, indicated that each farming household cultivates enset in its home garden. Planting and harvesting times in the enset agriculture system of the study area are not significantly different among the sample kebeles.

In Ethiopia, enset is produced mainly as a source of food for the family's subsistence [40]. In this study area, enset is also mainly produced as a source of food and also used for medicinal purposes, such as threatening ailments, feeding livestock, making fibber for house construction and material culture, and as a means of income generation. This study goes in line with the work of Shumbulo et al. [4] in the Offa district, Southern Ethiopia, in which enset is used as a source of food for the livelihood of the local community. In their study area, they are mainly used. For instance, corms, pseudostems, and stalks of the inflorescences were used in the form of ferments of the scraped leaf sheaths and grated corm mixed (kocho), a squeeze of the scraped leaf sheath, peduncle, and grated corm (bulla), and boiled enset corm (amicho). In this study, the various parts of enset, like a corm, pseudostem, and leaf, were used for medicinal purposes to treat human and livestock ailments. The local farmers replied that there was no known recommended dosage for the medical treatment of humans and livestock and that they should simply give the treatment until it becomes a cure for the disease. Thus, this research work has implications for enset crop conservation and the enhancement of the local people's livelihood. However, further research is needed to identify and distribute drought-tolerant agricultural landraces based on their climate adaptation for distribution, particularly to diverse parts of the country and hungry people in Sub-Saharan Africa.

## 5. Conclusions

This research confirms that local farmers' roles in the maintenance of their enset landraces are high. Farmers have their own indigenous enset planting material selection, multiplication, and management system. The identified enset landraces have morphological variability. Although some characters are the same throughout the study area, some other characters show some differences. Clustering similar landraces together and knowing morphological variation among landraces helps farmers select and maintain their landraces. Generally, the study indicated that the study area is rich in having different enset landraces, culture, and indigenous knowledge on enset production and maintenance. However, some endemic landraces have been lost in line for different reasons. As a result, the number of landraces will decrease, but some landraces and their habitats will become extinct, resulting in a loss of information, services, and cultural values related to enset crops, which could have long-term consequences.

## Figures and Tables

**Table 1:** The sample kebeles as well as the total number of respondents.

The name of the kebeles	Total household	Sample size proportion	The number of households in the sample
Ocha	661	0.32	46
Nekir	470	0.23	33
Mari Madara	541	0.26	38
Mari Guta	395	0.19	28
Total	2067	1.00	145

**Table 2:** The morphological characteristics of the study area's enset landraces.

Character	Code	Qualitative categories or quantitative measures
Pseudostem color	PSC	1 = light green, 2 = deep green, 3 = greenish black, 4 = light red, 5 = dark red, 6 = reddish yellow
Petiole color	PC	1 = light green, 2 = deep green, 3 = greenish black, 4 = light red, 5 = dark red, 6 = reddish yellow
Midrib color	MC	1 = light green, 2 = deep green, 3 = greenish yellow, 4 = greenish red, 5 = light red, 6 = dark red, 7 = dark brown
Leaf color	LC	1 = light green, 2 = deep green, 3 = light red, 4 = dark red, 5 = purple
Kocho quality	KQ	1 = high quality, 2 = medium quality, and 3 = low quality
Bulla quality	BQ	1 = high quality, 2 = medium quality, and 3 = low quality
Fiber quality	FQ	1 = high quality, 2 = medium quality, and 3 = low quality
Drought resistance	Dr.R	1 = resistance, 2 = venerable
Disease resistance	DR	1 = resistance, 2 = susceptible
Pseudostem length	PL	Meter
Pseudostem circumstances	Psc	Meter
Leaf length	LL	Meter
Leaf width	LW	Meter
Number of leaves	NL	Number
Plant height	HP	Meter

Note: Kocho is a fermented product made from scraped pseudostems and grated corm; bulla is a dehydrated juice.

**Table 3:** The various enset landraces.

S. no	Landraces' local names	Kebeles
1	Amiya	Ocha, Nekir
2	Yesha Maziya	Ocha, Nekir
3	Hoeya	Ocha, Guta
4	Bothena	Ocha, Nekir, Madara, Guta
5	Ontha	Ocha, Madara, Guta
6	Botha maziya	Ocha, Nekir, Guta
7	Erantiya	Ocha, Nekir, Madara, Guta
8	Shasha	Ocha
9	Ankuwa	Ocha
10	Boza	Ocha
11	Yaka	Ocha
12	Badadiya	Ocha
13	Argama	Ocha
14	Arkiya	Nekir
15	Budunthuwa	Nekir
16	Gena	Nekir
17	Shododiniya	Nekir
18	Mataka	Nekir
19	Aguntha	Nekir
20	Kuruwa	Guta
21	Wosa ayfiya	Guta
22	Keteriya	Guta

**Table 4:** The mean value of the quantitative characters for each enset landrace cluster.

Cluster	Mean plant height ± SD	Mean pseudo stem height ± SD	Mean pseudostem circumstances ± SD	Mean leaf width ± SD	Mean leaf length ± SD	Mean leaf number ± SD
Cluster 1	6.64 ± 0.87	2.65 ± 0.64	1.7 ± 0.30	0.53 ± 0.14	3.6 ± 0.7	14 ± 4.0
Cluster 2	7.7 ± 0.54	2.98 ± 1.13	2 ± 0.36	0.56 ± 0.15	4.33 ± 0.3	14 ± 4.4
Cluster 3	5.5 ± 0.282	2.2 ± 0.28	1.3 ± 0.14	0.65 ± 0.07	3.3 ± 0.6	22 ± 4.24
Cluster 4	7.3 ± 0.72	2.7 ± 0.46	1.67 ± 0.49	0.62 ± 0.06	4.47 ± 0.2	12 ± 5.73
Cluster 5	8.5 ± 0.897	3.07 ± 0.64	2.10 ± 0.37	0.62 ± 0.15	4.63 ± 0.6	15 ± 5.28

**Table 5:** The richness (*C*), evenness (*E*), Simpson (*D*), and Shannon (*H*) indices were used to assess the diversity of landraces in the study area.

Kebele	Richness (C)	Diversity index (H)	Evenness
Ocha	17	2.28	1
Nekir	14	2.24	1
Mari Guta	9	2.17	0
Mari Madara	16	2.22	1

**Table 6:** A direct matrix ranking of ten Mareka district landraces against eight properties, with three indicating the best, two indicating the medium, and one indicating the worst.

Properties	Landraces									
	Amiya	Yesha Maziya	Boza	Hoeya	Bothena	Ontha	Ankuwa	Shasha	Yaka	Botha Maziya
Yield	1.40	1.40	2.80	2.40	1.40	2.60	2.25	2.50	1.40	2.90
Maturity time	1.60	1.60	1.50	2.20	2.80	2.60	2.60	3.00	1.40	1.25
Taste	2.50	1.40	1.80	1.50	1.25	2.25	2.50	1.50	2.80	2.80
Drought tolerance	1.25	2.20	2.60	2.50	2.50	1.40	2.80	1.50	1.60	3.00
Disease resistance	2.60	2.30	2.80	2.25	2.50	2.60	2.80	1.12	1.40	2.80
Kocho quality	1.60	1.25	2.40	2.50	2.60	2.90	2.60	1.40	1.75	2.80
Bulla quality	1.40	1.40	2.50	2.40	2.50	2.90	2.60	2.40	2.60	2.90
Fiber quality	2.60	2.25	3.00	1.90	2.50	2.60	2.60	1.60	1.50	2.60
Total	14.75	13.75	19.40	17.80	19.15	19.80	20.75	15.02	14.45	21.05
Rank	8^th^	10^th^	4^th^	6^th^	5^th^	3^rd^	2^nd^	7^th^	9^th^	1^st^

**Table 7:** Eating habits and food preparation methods for enset.

Landraces name	Types of food	Methods of preparation
All the landraces	Kocho	Scrapped leaf sheath and grated corm mix, wrapped in enset and stored underground until fermented
All the landraces	Bulla	Scraping the leaf sheath peduncle, grating the corm into a pulp, and squeezing liquid containing starch from the pulp, allowing the resultant starch to concentrate into white powder and rehydrating with water

**Table 8:** Enset landraces, part/s used for treatment, disease type, medicinal use, and preparation methods.

The name of the landrace	Parts used	Used to treat disease or injury	Methods of preparation
Arkiya	Corm	To cure a cough; to dry an abscess; to restore normal body function	The corm is boiled and eaten with milk
Lochingiya	Corm	Used to join the broken body (bone), for lung disease and cough, to harden the damaged organ	The corm is boiled and eaten with cheese
Badadiya	Corm and pseudostem	To repair and soften the broken body (bone) and initiate milk production for the mammary gland of the woman	The corm is sliced and boiled, and the starchy powder, bulla, is eaten with milk
Kataniya	Corm and pseudostem	To dry the wounds of humans and cattle	The corm is boiled and given to cattle with salt
Boza	Corm and pseudostem	For fattening of livestock For the normal functioning of the body	Corticated and given to the livestock
Argama	Corm	For the normal functioning of the body	The corm is boiled and eaten with milk
Aguntha	Corm	Used to join the broken body (bone), for lung disease and cough, to harden the damaged organ	The corm is boiled and eaten with cheese
Mataka	Corm and pseudostem	To repair and soften the broken body (bone) and initiate milk production for the mammary gland of the woman	The corm is sliced and boiled, and the starchy powder, bulla, is eaten with milk

**Table 9:** Preference ranking for the use-value of enset.

Data collection kebele	Food	Feed	Medicinal	Fiber	Income generation
Ocha	5	4	3	3	2
Nekir	5	4	2	4	3
Guta	5	3	3	2	1
Madara	5	2	2	3	2
Total	20	13	10	12	8
Rank	1^st^	2^nd^	4^th^	3^rd^	5^th^

## Data Availability

Data sharing is not applicable to this manuscript as no datasets were generated or analyzed during the current study.
